# Exploring the influence from whole blood DNA extraction methods on Infinium 450K DNA methylation

**DOI:** 10.1371/journal.pone.0208699

**Published:** 2018-12-12

**Authors:** Hanne Sagsveen Hjorthaug, Kristina Gervin, Petter Mowinckel, Monica Cheng Munthe-Kaas

**Affiliations:** 1 Department of Medical Genetics, Oslo University Hospital and University of Oslo, Oslo, Norway; 2 PharmaTox Strategic Research Initiative, Faculty of Mathematics and Natural Sciences, University of Oslo, Oslo, Norway; 3 Pharmacoepidemiology and Drug Safety Research Group, Department of Pharmacy, School of Pharmacy, University of Oslo, Oslo, Norway; 4 Department of Pediatric and Adolescent medicine, Oslo University Hospital, Oslo, Norway; 5 Department of Pediatric Oncology and Hematology, Oslo University Hospital, Oslo, Norway; Centre de Recherche en Cancerologie de Lyon, FRANCE

## Abstract

Genome-wide DNA methylation studies are becoming increasingly important in unraveling the epigenetic basis of cell biology, aging and human conditions. The aim of the present study was to explore whether different methods for extracting DNA from whole blood can affect DNA methylation outcome, potentially confounding DNA methylation studies. DNA was isolated from healthy blood donors (n = 10) using three different extraction methods (i.e. two automatic extractions methods based on magnetic beads or isopropanol precipitation, and manual organic extraction). DNA methylation was analyzed using the Infinium HumanMethylation450 Bead Chip (Infinium 450K) (n = 30 samples in total), which is a frequently used method in genome-wide DNA methylation analyses. Overall, the different extraction methods did not have a significant impact on the global DNA methylation patterns. However, DNA methylation differences between organic extraction and each of the automated methods were in general larger than differences between the two automated extraction methods. No CpG sites or regions reached genome-wide significance when testing for differential methylation between extraction methods. Although this study is based on a small sample, these results suggest that extraction method is unlikely to confound Infinium 450K methylation analysis in whole blood.

## Introduction

Epigenetic mechanisms such as DNA methylation and histone modifications have important roles in human biology and diseases (e.g. cell differentiation [1], x-inactivation [2;3] and gene expression [4;5]). In mammals, DNA methylation is found mainly at CpG sites, and is highly variable between individuals, cell-types, and tissues [6]. DNA methylation is the most widely studied epigenetic modification in humans, and Epigenome-wide association studies (EWAS) are commonly used to investigate the association between DNA methylation variation and a range of phenotypes (e.g. clinical parameters [7;8] or disease status [9;10]).

Today, researchers have access to various sources of biological material, which are frequently used in EWAS. However, the method used to extract DNA often differs across samples in a study, reflecting e.g. different tissue types, different laboratories involved or changes in procedures over time. Consequently, it is important to know whether DNA extraction method can affect DNA methylation outcome and be a confounding factor in downstream analyses. The aim of the present study was to determine whether three different DNA extraction methods (automatic extractions on MagNA Pure LC and Autopure LS, and manual organic extraction) could influence Infinium 450K DNA methylation values in whole blood. The choice of extraction methods was based on procedures previously used for in-house biobanking, and rely upon three different principles frequently applied in commercial kits and custom protocols (magnetic beads, salting-out followed by isopropanol precipitation, and organic extraction).

## Materials and methods

### Subjects and DNA extraction methods

Peripheral whole blood samples from anonymous healthy blood donors (n = 10) were collected at the Blood Bank at Oslo University Hospital. DNA was extracted from each donor applying three different extraction methods: 1) automatic extraction with a magnetic bead-based procedure on MagNA Pure LC (Roche Diagnostics), 2) automatic extraction with salting-out and isopropanol precipitation on Autopure LS (Qiagen), and 3) manual organic extraction followed by ethanol precipitation. All extractions were done with an input of 1 mL blood. Blood was stored at 4°C prior to DNA extraction, with storage time being 2/24/5 hours for MagNA Pure/Autopure/organic extractions, respectively. Final DNA concentrations were measured using Quant-iT dsDNA High-Sensitivity Assay kit on the Qubit fluorometer (Invitrogen). DNA quality was assessed from NanoDrop 8000 spectrophotometer measurements (Thermo Scientific).

The automatic DNA extractions were performed according to manufacturer’s instructions. The manual DNA extraction was performed as follows: 4 mL lysis buffer (0.32 M sucrose, 10 mM Tris HCl pH 7.6, 5 mM MgCl_2_, 1% Triton-X-100) was added to 1 mL blood in a 10 mL centrifuge vial and mixed for 10 min at 4°C in a tabletop shaker. The vial was centrifuged at 400 x g for 15 min (4°C) and the supernatant was discarded. 2 mL PBS was added to the pelleted nuclei, followed by another centrifugation at 400 x g for 15 min (4°C). The supernatant was discarded and the pellet was resuspended by vortexing in 200 μl of a cold buffer containing 75 mM NaCl and 25 mM EDTA pH 8.0. 20 μl 10% SDS was added and the vial swirled to mix. The sample was transferred to a 1.5 mL tube, 2.2 μl 10 mg/mL proteinaseK was added and the tube swirled to mix. The sample was incubated at 55°C for 90 min with shaking (1200rpm) in a tabletop thermomixer. DNA was isolated from the sample by standard phenol/chloroform extractions followed by ethanol precipitation (protocol available upon request).

### DNA methylation analysis

#### Bisulfite conversion

1 μg of DNA was bisulfite converted using EZ DNA Methylation kit (Zymo Research), using incubation conditions recommended for Infinium 450K samples. All samples were converted in the same batch.

#### Preprocessing and quality control

DNA methylation was assessed using the Infinium HumanMethylation450 BeadChip (Illumina). The three extracted DNA samples from each individual were run on the same BeadChip to minimize potential batch effects. Data were preprocessed using the approach implemented in the *RnBeads* package [11]. Background subtraction was performed using methylumi.noob and β-values were normalized using BMIQ. Cross-reactive probes (n = 30,969), poor quality probes (n = 1,644, applying the Greedycut algorithm to remove probes with detection p-value > 0.01), non-CpG probes (1,385), and probes with missing values (n = 1,146) were removed. For each individual, gender was predicted by inspecting methylation density plots of the X and Y chromosomes, revealing a 50/50% male/female distribution. In order to keep only those sites having reliable measurements from all ten individuals for downstream analyses, probes on sex-chromosomes were removed (n = 10,323).

The preprocessed data set is deposited in Zenodo (DOI 10.5281/zenodo.1285774). Raw data is available upon request.

#### Bland-Altman analysis

This, and all other statistical analyses, were carried out using the R programming language [12].

Pairwise comparisons of the different DNA extraction methods (MagNA Pure versus Autopure (MvsA), MagNA Pure versus organic extraction (MvsO), and Autopure versus organic extraction (AvsO)) were performed using a modified version of the method described by Bland and Altman [13]. For all comparisons, a Bland-Altman (B-A) plot was constructed, in which each of the 4,401,100 average intra-individual DNA methylation values (10 subjects of 440,110 values each) was plotted against its corresponding DNA methylation difference. In the original method by Bland and Altman, limits of agreement are drawn in the B-A plot as horizontal lines showing the mean difference ±1.96 times the standard deviation of the differences. However, this approach assumes differences to be normally distributed, which is not satisfied by our data set (non-normality confirmed for each pairwise comparison by ten repeated Shapiro-Wilk tests on random samples (n = 5,000)). Inspection of our B-A plots indicates a god fit for a regression model of the type *diff*_*i*_ *~ σ(x*_*i*_*)e*_*i*_ for i = 1,…,n, with *diff*_*i*_ being DNA methylation difference number i, *x*_*i*_ denoting its corresponding average DNA methylation value, each *e*_*i*_ ~ N(0,1), and the variable standard deviation described by a function of the type *σ(x) = e*^*{a+b(x-0*.*5)^2}*^. Constants a and b were estimated using non-linear minimization, and limits of agreement was drawn as +/- 1.96·σ(x) for each Bland-Altman plot.

#### Principal component analysis

Principal component analysis (PCA) was performed to examine the data set for strong signals related to extraction method. A non-parametric Kruskal-Wallis test was used to test for association between each PC and extraction method, and between PCs and sample position on chip. To test for association between PCs and per sample DNA yield, and between PCs and per sample DNA quality measure (A260/A280, and A260/A230), two-sided Spearman’s correlation tests were performed. Adjustment for multiple testing was done using Bonferroni correction, and corrected p-values < 0.05 was considered significant.

#### Differential DNA methylation analysis

Per pairwise method comparison (MvsA, MvsO, AvsO), a paired t-test was performed to test for difference in global methylation, with significance level set to 0.05.

Also, per pairwise method comparison, paired t-tests were performed to search for differentially methylated CpGs between methods. A false discovery rate (FDR) cutoff of less than 5% was used for genome-wide significance by applying the method of Benjamini and Hochberg (BH) [14].

The *BumhunterEngine* function in the Bioconductor package *Bumphunter* [15;16] was used to search for differentially methylated regions between methods. Clusters were made using the *ClusterMaker* function (maxGap = 1000). *BumphunterEngine* arguments were set as follows: cutoff = 0.01, number of bootstraps = 250, and *loessByCluster* for smoothing. To adjust for multiple testing, a family-wise error rate (fwr) cutoff less than 5% was used for genome-wide significance.

#### Analyses on predicted age and cell type proportions

Age and cell type proportions (CD8+ and CD4+ T-lymphocytes, natural killer cells, B cells, monocytes, and granulocytes) were estimated for each sample (subject 1–10, three extraction methods each) using the DNA Methylation Age Calculator [17], applying a Houseman reference-based approach and a peripheral adult blood reference data set [18]. Per pairwise method comparison (MvsA, MvsO, AvsO), paired Wilcoxon tests were performed to test for differences in predicted age and cell type proportions. Adjustment for multiple testing was done using Bonferroni correction, and corrected p-values < 0.05 were considered significant.

## Results

### DNA extractions

DNA yield were on average 16 μg for the MagNA Pure extractions, 7 μg for the Autopure extractions, and 17 μg for the manual organic extractions (Table 1). Autopure extracted samples showed in general somewhat low A260/A230 ratios, indicating leftover isopropanol/ethanol after the final precipitation and washing steps. Otherwise, A260/A280 and A260/A230 ratios were good for all samples.

**Table 1:** DNA concentrations and purity measures.

	Subject	Yield (μg)	A260/280	A260/230
**MagNA Pure**	1	18	1.93	1.94
	2	17	2.00	2.06
	3	31	1.93	2.08
	4	18	1.96	1.91
	5	11	1.95	1.95
	6	15	1.98	2.05
	7	12	1.95	1.98
	8	13	1.99	2.04
	9	16	1.89	2.11
	10	14	2.00	2.14
**Autopure**	1	3	1.98	0.93
	2	6	1.92	1.58
	3	17	1.94	1.66
	4	2	1.89	1.12
	5	4	1.90	1.41
	6	2	1.95	1.27
	7	4	1.94	1.32
	8	10	1.93	1.78
	9	19	1.91	2.05
	10	6	1.89	1.55
**Manual organic extraction**	1	8	1.92	2.10
	2	27	1.94	2.33
	3	15	1.94	2.32
	4	9	1.95	2.29
	5	8	1.93	2.37
	6	13	1.92	2.31
	7	21	1.91	2.32
	8	18	1.93	2.41
	9	36	1.90	2.42
	10	19	1.91	2.60

### General description of the data

Overall, DNA methylation differences between methods were very small, with 99% or more of the CpG sites showing a mean methylation difference lower than 0.03 (Table 2). DNA methylation differences between organic extraction and each of the automated methods were skewed towards slightly larger values as compared to differences between the two automated extraction methods.

**Table 2:** Distributions of mean DNA methylation differences between the three extraction methods.

DNA methylation difference	MagNA Pure vs Autopure	MagNA Pure vs organic extraction	Autopure vs organic extraction
**0.00–0.01**	90.5	83.1	84.1
**0.01–0.02**	8.3	13.1	12.1
**0.02–0.03**	1.0	2.9	2.8
**0.03–0.04**	0.20	0.69	0.70
**0.04–0.05**	0.05	0.18	0.21
**0.05–0.06**	0.015	0.059	0.066
**0.06–0.07**	0.004	0.018	0.019
**0.07–0.08**	0.001	0.007	0.007
**0.08–0.09**	0.002	0.003	0.002
**0.09–0.10**	0.001	0.001	0.001
**>0.10**	0.001	0.002	0.001

For each method comparison, the numbers represent the percentages of sites (out of the 440110 studied sites) observed for different ranges of absolute mean DNA methylation differences across samples (n = 10).

**Fig 1:**
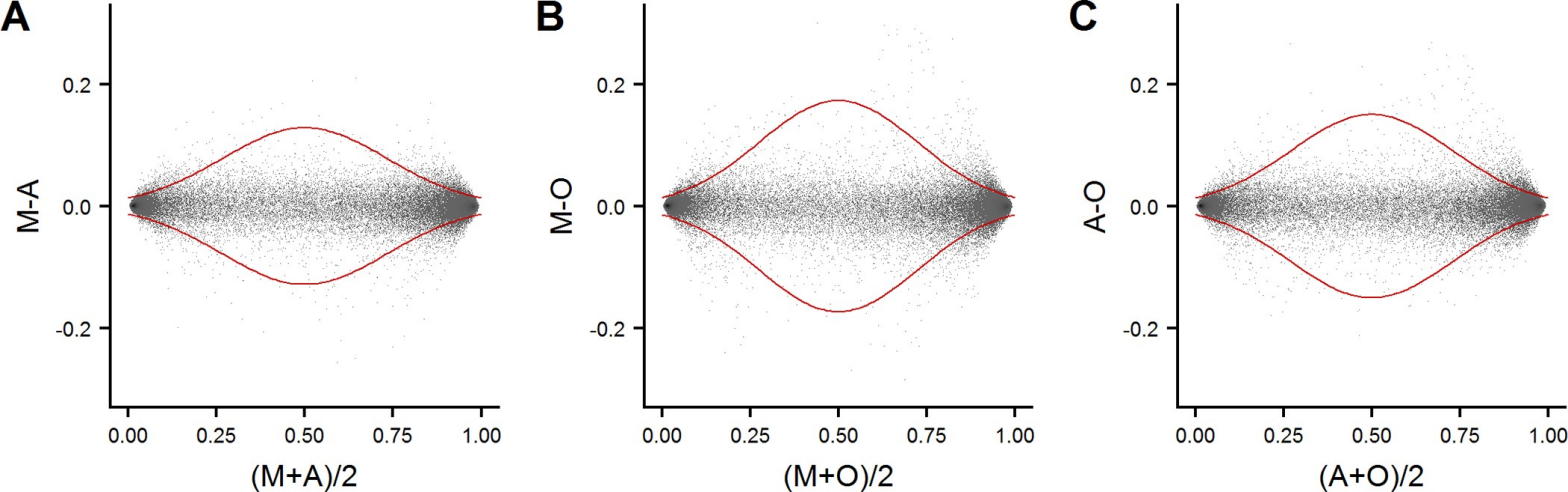
Bland-Altman plots. Bland-Altman plots of pairwise comparisons of the three extraction methods. Each plot is a visualization of the 4,401,100 mean intra-individual DNA methylation values (10 subjects of 440,110 values each, on the x-axis) plotted against their corresponding DNA methylation differences on the y-axis (brightest shade corresponds to one observation per point, while black shade corresponds to 400 observations per point). Limits of agreement are drawn in red. **A)** MagNA Pure extracted DNA (M) vs Autopure extracted DNA (A). **B)** MagNA Pure extracted DNA (M) vs organic extracted DNA (O). **C)** Autopure extracted DNA (A) vs organic extracted DNA (O).

To allow for visual judgement of the agreement between pairs of methods, we applied Bland-Altman analyses to the data set (Fig 1). As depicted, there was no major skewness in these plots. The widths of limits of agreement suggest that the agreement is better between the two automated extraction methods than between organic extraction and each of the automated methods.

**Fig 2:**
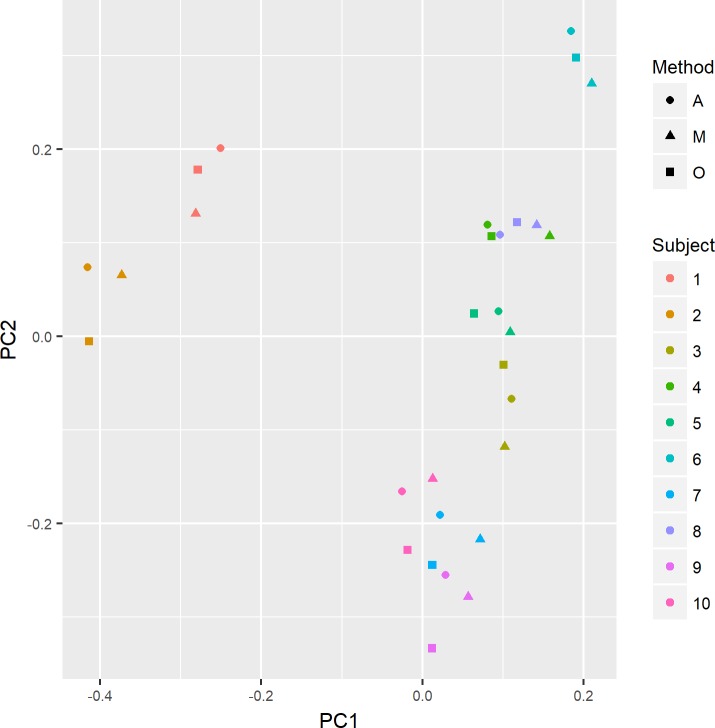
PCA plot of DNA methylation for all samples. DNA methylation values for each sample (n = 30) are represented by PC1 and PC2, which together explain 37% of the variance between samples.

The relationship between methylation differences and DNA extraction methods was explored further, applying PCA on the data set. A plot of the first two PCs is shown in Fig 2. Here, the extraction methods for each individual cluster together. This shows that biological differences are substantially larger than differences related to extraction methods. When testing for association between PCs and extraction methods, PC 12 was found to be significantly associated (p = 0.021), explaining 1.9% of the variance in the data set. Sample position on BeadChip, DNA yield, A260/A280 ratio, and A260/A230 ratio were not significantly associated to any of the principal components.

### No significant differences associated with DNA extraction at the global, regional or CpG level

Next, we investigated whether there were differences between pairs of extraction methods at the global, regional or CpG level. There were no significant difference in global DNA methylation between methods; neither did the analyses reveal differentially methylated regions or CpG sites.

### No significant differences in predicted age and cell type proportions between DNA extraction methods

**Fig 3:**
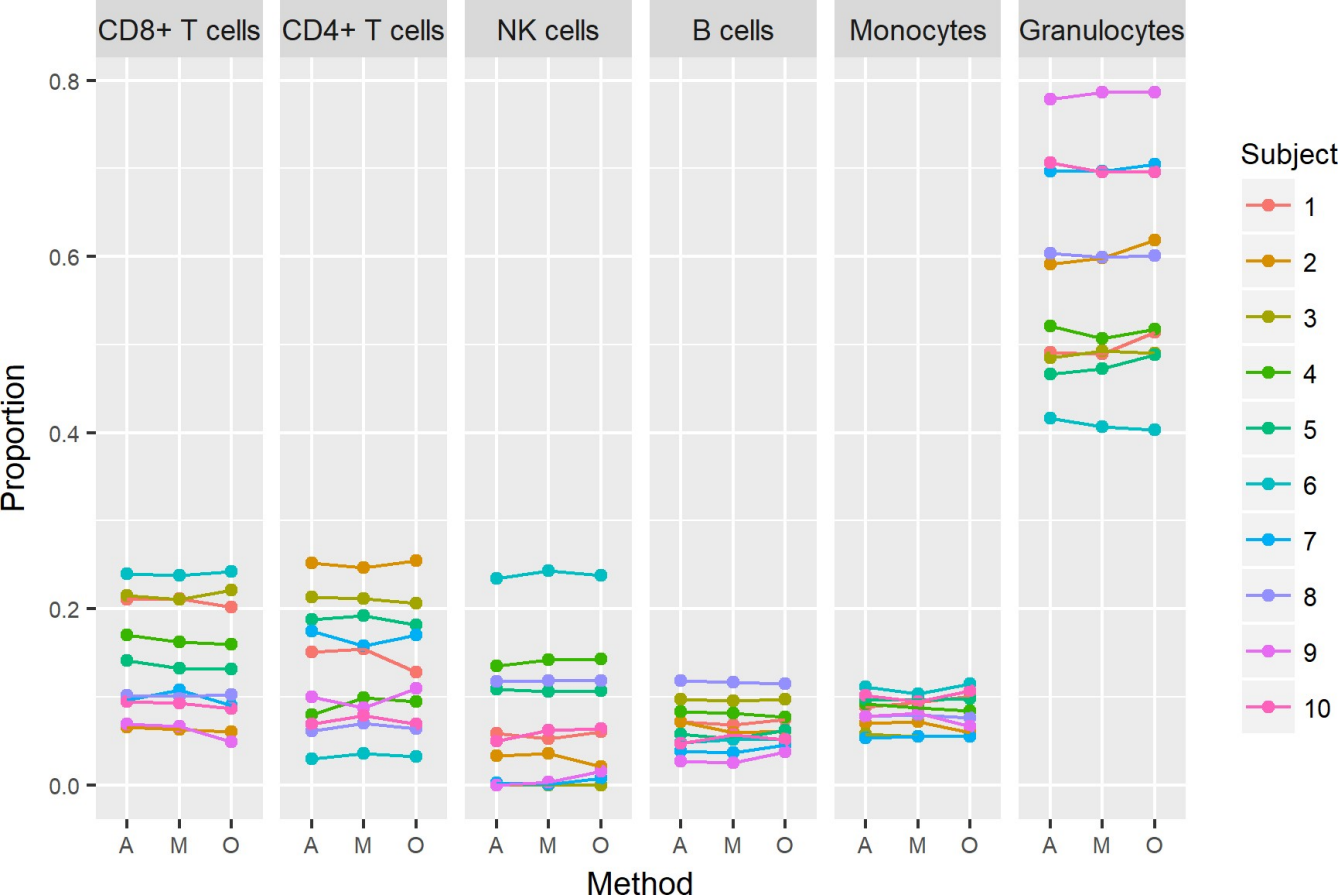
Estimated cell type proportions. Estimated cell type proportions for each sample (subject 1–10, three extraction methods each (A, M, O)).

Estimated cell type proportions are shown in Fig 3. Our analyses did not reveal any significant differences in predicted cell type proportions between DNA extraction methods, or any differences in predicted age.

## Discussion

In the present study we have shown that differentially extracted DNA exhibit small differences in Infinium 450K DNA methylation. Manual organic DNA extraction seemed to introduce slightly more variation in DNA methylation compared to automatic extraction methods. We did not find evidence that different DNA extraction methods will confound downstream differential DNA methylation analysis at the global, regional, or CpG level. In line with this, we show that prediction of age and cell type proportions are replicated across the methods.

Out of the DNA extraction methods applied, Autopure extractions gave clearly less DNA, and lower A260/230 ratios, than the other two methods. Despite these differences we did not find DNA yield or quality measures to be associated to the principal components of the data set, neither were there any differences between the methods regarding the number of unreliable measurements (detection p-value > 0.01) in the data set (data not shown).

To our knowledge, this is the first study exploring whether DNA extraction method generates differences in DNA methylation using Infinium 450K. Shiwa *et al*. [19] have explored the influence on Infinium 450K values from different blood collection protocols, but this study was not designed to differentiate between effects caused by blood storage conditions and effects caused by DNA extraction methods. They found that estimated cell-type proportions differed between collection protocols, and concluded that correcting for cell-type composition minimized systematic bias in the DNA methylation profiles. It is worth noticing that their statistical analysis included a correction for technical bias, which may diminish the effects from DNA extraction method. Thus, the observed differences in cell-type proportions could be mediated through different storage conditions alone. In a separate experiment Shiwa *et al*. demonstrated that a 24h storage period at 4°C prior to buffy coat isolation, followed by DNA extraction, led to changes in cell-type compositions. We did not observe any significant differences in cell-type proportions estimated from whole blood extracted DNA, despite differences in blood storage time at 4°C (Fig 3).

The results of the present study should be interpreted in light of several limitations and strengths. The design enabled paired analyses of the DNA extraction methods, excluding confounding by covariates such as genetics and age. Further, the methods are based upon different principles (magnetic beads, salting-out followed by isopropanol precipitation, and organic extraction) that are used in many commercial kits and custom protocols. Consequently, our results should be relevant for a wide range of DNA isolation procedures. Also, Infinium 450K is a widely used DNA methylation assay, recently replaced by Illumina’s EPIC array. The EPIC array relies on the same chemistry as Infinium 450K, and more than 90% of the Infinium 450K content is to be found on this array. Thus, our results will be of importance for evaluation of Infinium 450K results, and for researchers planning to run EPIC arrays.

A limitation of our study is that we cannot differentiate between factors that are directly or indirectly connected to DNA extraction method. Although assigned to the DNA extraction step, putative effects do not necessarily rely on the different extraction procedures by themselves, but could be due to factors such as laboratory temperature and humidity. A more complex study design would have been required to control for such factors.

Given the minor variation in DNA methylation observed between the methods, the main limitation of our work is the small sample size, and subsequent poor power to detect small effect sizes. Thus, we can not exclude DNA extraction method as a confounder in studies exploring small effect sizes. In an exploratory approach we looked into the top hits from the t-tests for differentially methylated CpGs (albeit non-significant), and found indications that MagNA Pure extraction might slightly underestimate, while organic extraction might slightly overestimate, Infinium 450K DNA methylation (Figures in S1–S3 Figs). We found the putative effect from organic extraction on DNA methylation to be skewed towards open sea and repetitive elements (data not shown). Due to rapid strand reannealing, repetitive elements are expected to have a high rate of false positive 5-methylcytosines following bisulfite conversion [20]. We hypothesize that organic extraction exaggerates this tendency, possibly through salt leftovers increasing DNA melting temperatures [21;22].

DNA methylation studies rely on high quality material not associated with technical variation, which could confound down-stream analysis. Several issues could be addressed concerning whether DNA samples are suitable for DNA methylation analysis, and whether it is appropriate to compare samples from different batches or cohorts. In the present work we have shed light on the use of different DNA extraction methods prior to DNA methylation analysis. The results presented here show that the three extraction methods included in this study did not introduce significant changes in whole blood DNA methylation outcome at the global, regional or CpG level, as measured by Infinium 450K.

## Supporting information

S1 Fig Distribution of CpG sites according to t-test p-value and sign of the methylation difference. Distribution of CpG sites according to p-value from t-test for differentially methylated CpGs, and to sign of the methylation difference, is given for three p-value cutoffs: p < 0.002, p < 0.01, and p > 0.75. “M>A”: MagNA Pure extracted DNA holds the higher mean methylation measure in the MagNA Pure vs Autopure comparison (MvsA); “M<A”: MagNA Pure extracted DNA holds the lower mean methylation measure in the MvsA comparison; and so on for MagNA Pure vs organic extraction (MvsO) and Autopure vs organic extraction (AvsO). Proportions are given as percentages of sites in each p-value cutoff group. The number of sites in each group is n = 5182, n = 24205, and n = 262234, for p-value cutoffs of < 0.002, < 0.01, and > 0.75, respectively.
(TIF)

S2 Fig Distribution of CpG sites according t-test p-value and between-method ranking of methylation values. Distribution of CpG sites according to p-value from t-test for differentially methylated CpGs, and to between-method ranking of methylation values (lowest or highest value), is given for three p-value cutoffs: p < 0.002, p < 0.01, and p > 0.75. “M lowest”: MagNA Pure extracted DNA holds the lower mean methylation measure out of the three extraction methods, and p-value cutoff is valid for both MvsA and MvsO tests for differentially methylated CpGs; “M highest”: MagNA Pure extracted DNA holds the higher mean methylation measure out of the three extraction methods, and p-value cutoff is valid for both MvsA and MvsO tests for differentially methylated CpGs; and so on for A and O. Proportions are given as percentages of sites in each p-value cutoff group. The number of sites in each group is n = 235, n = 2389, and n = 34775, for p-value cutoffs of < 0.002, < 0.01, and > 0.75, respectively.
(TIF)

S3 Fig Mean DNA methylation differences of CpG sites according to t-test p-value and between-method ranking of methylation values. Boxplots of absolute mean DNA methylation differences across subjects (n = 10) for the CpG sites in “M lowest” and “O highest” groups in S2 Fig.
(TIF)

S1 Document Data protection officer statement. Statement regarding data sharing from the Data Protection Officer at Oslo University Hospital.
(DOCX)
