# Supplementary figures and images for: Exploring the influence from whole blood DNA extraction methods on Infinium 450K DNA methylation
Source: PLoS One. 2018 Dec 12;13(12):e0208699. doi: 10.1371/journal.pone.0208699 (PMC6291135; doi:10.1371/journal.pone.0208699)

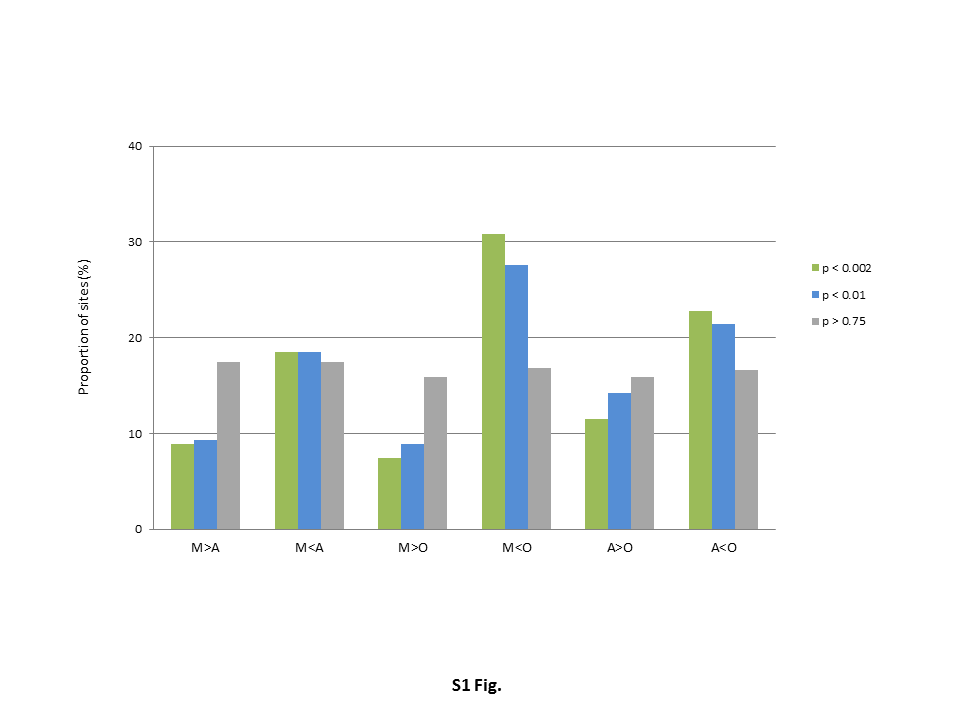

Supplement: S1 Fig — Distribution of CpG sites according to p-value from t-test for differentially methylated CpGs, and to sign of the methylation difference, is given for three p-value cutoffs: p < 0.002, p < 0.01, and p > 0.75. “M>A”: MagNA Pure extracted DNA holds the higher mean methylation measure in the MagNA Pure vs Autopure comparison (MvsA); “M<A”: MagNA Pure extracted DNA holds the lower mean methylation measure in the MvsA comparison; and so on for MagNA Pure vs organic extraction (MvsO) and Autopure vs organic extraction (AvsO). Proportions are given as percentages of sites in each p-value cutoff group. The number of sites in each group is n = 5182, n = 24205, and n = 262234, for p-value cutoffs of < 0.002, < 0.01, and > 0.75, respectively. (TIF) [file pone.0208699.s001.tif]

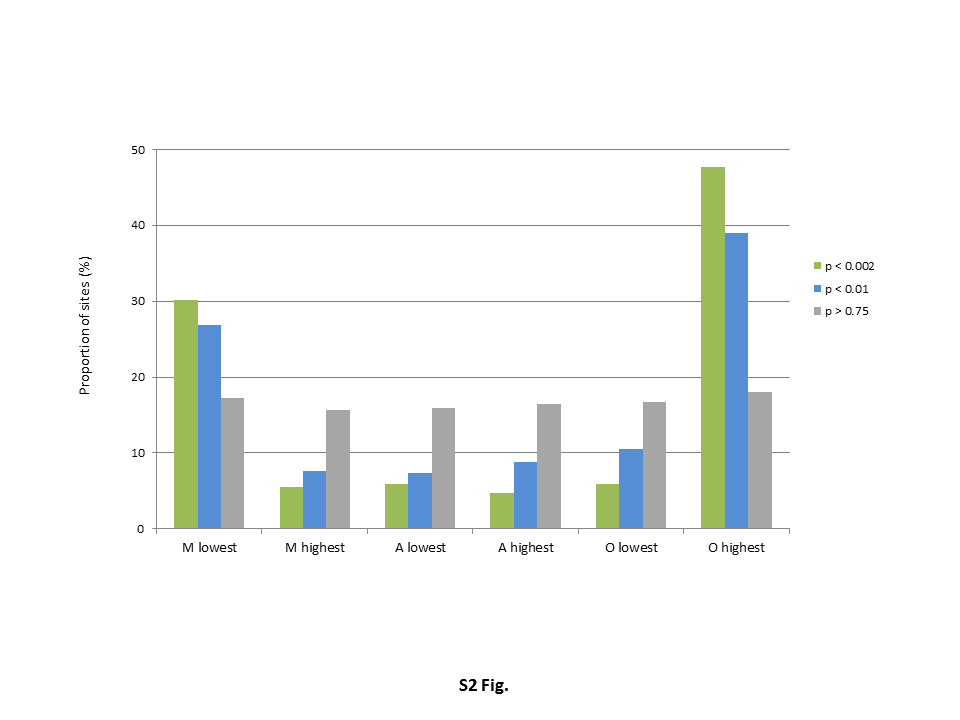

Supplement: S2 Fig — Distribution of CpG sites according to p-value from t-test for differentially methylated CpGs, and to between-method ranking of methylation values (lowest or highest value), is given for three p-value cutoffs: p < 0.002, p < 0.01, and p > 0.75. “M lowest”: MagNA Pure extracted DNA holds the lower mean methylation measure out of the three extraction methods, and p-value cutoff is valid for both MvsA and MvsO tests for differentially methylated CpGs; “M highest”: MagNA Pure extracted DNA holds the higher mean methylation measure out of the three extraction methods, and p-value cutoff is valid for both MvsA and MvsO tests for differentially methylated CpGs; and so on for A and O. Proportions are given as percentages of sites in each p-value cutoff group. The number of sites in each group is n = 235, n = 2389, and n = 34775, for p-value cutoffs of < 0.002, < 0.01, and > 0.75, respectively. (TIF) [file pone.0208699.s002.tif]

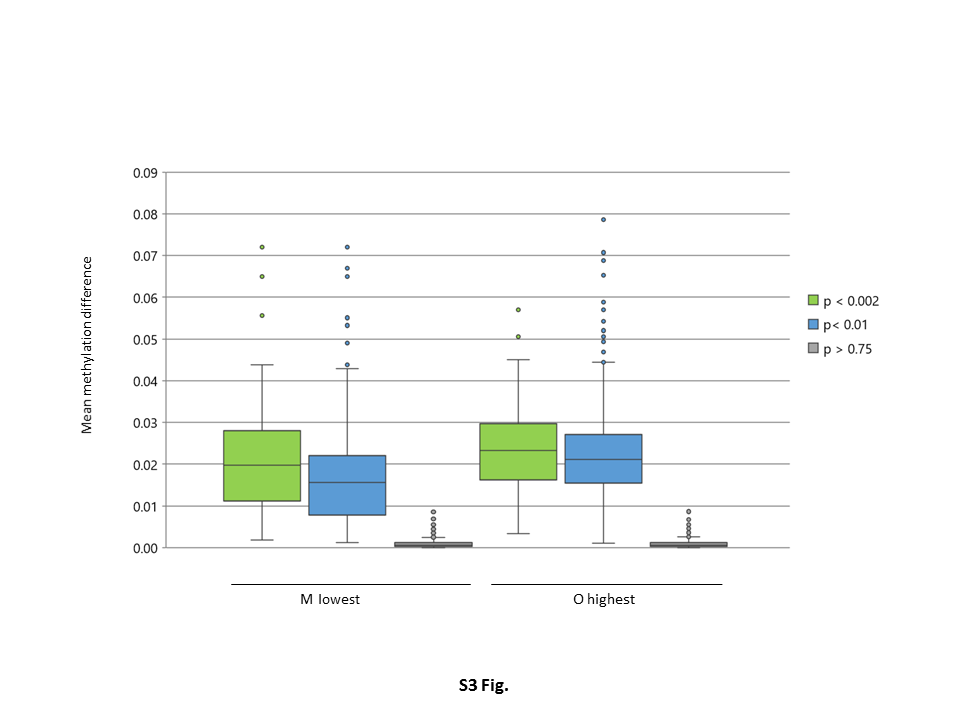

Supplement: S3 Fig — Boxplots of absolute mean DNA methylation differences across subjects (n = 10) for the CpG sites in “M lowest” and “O highest” groups in S2 Fig. (TIF) [file pone.0208699.s003.tif]
