# Supplementary material for: Exploring the influence from whole blood DNA extraction methods on Infinium 450K DNA methylation
Source: PLoS One. 2018 Dec 12;13(12):e0208699. doi: 10.1371/journal.pone.0208699 (PMC6291135; doi:10.1371/journal.pone.0208699)
Supplement: S1 Document — Statement regarding data sharing from the Data Protection Officer at Oslo University Hospital. (DOCX) [file pone.0208699.s004.docx]

|  | | | |  | | | | | |
| --- | --- | --- | --- | --- | --- | --- | --- | --- | --- |
| 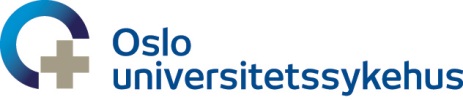 | | | | | **Oslo universitetssykehus HF** | | |  |  |
|  | | | | | Postadresse:  Postboks 4950 Nydalen  0424 Oslo  Sentralbord:  02770  Org.nr:  NO 993 467 049 MVA  www.oslo-universitetssykehus.no | | |  |  |
| **Data Protection Officer**  **statement** | | | | |  |  |  |  |  |
| Til: | Monica Cheng Munthe-Kaas,  Chief Attending Physician  BAR BARNEMEDISINSK AVDELING | | | |  | | | |  |
|  |  | | | |  |  |  |  |  |
| Fra: | Data Protection Officer, Oslo University Hospital | | | |  |  |  |  |  |
| Saksbehandler: | Tor Åsmund Martinsen | | | |  |  |  |  |  |
| Dato: | 24.10.18 | | | |  |  |  |  |  |
|  |  | | | |  | | | |  |

**The Data Protection Officer statement in processing personal data regarding**

**Study: Exploring the influence from whole blood DNA extraction methods on Infinium 450K DNA methylation**

Purpose: This is a quality study exploring the effects of DNA extraction methods on DNA methylation.

Duration: June-July 2012

This study is based on data generated from 10 anonymous blood donors. According to our regulations, it is not defined as health research, and hence there are no requirements for ethical permission or approval from the Data Protection Officer. Nevertheless, the raw dataset underlying the findings in the present work has been considered potentially identifiable in a future perspective. Thus, our institutional policy does not allow the raw dataset to be shared publicly. The raw dataset can be shared with other researchers upon request, following a data sharing agreement.

Best regards

Tor Åsmund Martinsen

Data Protection Adviser

Oslo University Hospital HF

Staff Patient Safety and interaction

Department of Information Security and Data Protection
